# Rheological and Aging Properties of Nano-Clay/SBS Composite-Modified Asphalt

**DOI:** 10.3390/ma17174376

**Published:** 2024-09-04

**Authors:** Yeqing Lu, Siwei Li, Yixin Jiang, Xiaolong Yang, Linxianzi Li

**Affiliations:** 1 Guangxi Jiaotou Technology Co., Ltd., Nanning 530000, China; luyq@bgigc.com; 2 School of Civil Engineering and Architecture, Guangxi University, Nanning 530004, China; siweili@st.gxu.edu.cn (S.L.); 2310391051@st.gxu.edu.cn (L.L.); 3 Guangxi Communications Investment Group Guilin Expressway Operation Co., Ltd., Guilin 541100, China; jyx16030423@163.com

**Keywords:** modified asphalt, OMMT/SBS, micro mechanism, rheological properties, aging resistance

## Abstract

Nano-organic montmorillonite (OMMT) not only inhibits the harmful asphalt fume generation during the production and construction processes of asphalt mixtures but also effectively improves the performance of asphalt pavements. In order to prepare asphalt materials with smoke suppression effects and good road performance, this study selects nano-OMMT and SBS-modified asphalt for composite modification of asphalt mixtures and systematically investigates its road performance. Through the temperature sweep test, the frequency sweep test, the multiple stress creep recovery (MSCR) test, the bending beam rheometer (BBR) test, and the atomic force microscope (AFM) test, the high-temperature rheological properties, low-temperature rheological properties, high-temperature properties and aging resistance of the modified asphalt are studied. The research findings indicate that OMMT can effectively reduce the sensitivity of modified asphalt to load stress and improve its high-temperature rheological properties. SBS-modified asphalt shows increased creep stiffness and a decreased creep rate after OMMT modification, resulting in reduced flexibility and decreased low-temperature crack resistance. After short-term and long-term aging, the complex modulus aging index of OMMT/SBS composite-modified asphalt is lower than that of SBS-modified asphalt, and the phase angle aging index is higher than that of SBS-modified asphalt, demonstrating that OMMT enhances the aging resistance of SBS-modified asphalt. OMMT inhibits oxidation reactions in the asphalt matrix, reducing the formation of C=O and S=O bonds, thereby slowing down the aging process of modified asphalt and improving its aging resistance.

## 1. Introduction

In recent years, China’s road mileage has continually reached new highs, with an increasing proportion of high-grade roads. By the end of 2022, the total length of roads in China had reached 5.35 million kilometers, with expressways totaling 177,000 km. Cement concrete pavement is used in traditional highway pavements, which has issues such as high stiffness, difficult maintenance, and poor skid resistance. However, asphalt pavements offer advantages like shorter construction times, easier maintenance, better vibration and noise reduction, smoothness, and good skid resistance, making them widely used in the road engineering industry. Currently, traditional hot mix asphalt (HMA) is typically used in asphalt pavement construction operations, which releases a significant amount of harmful emissions during high-temperature transportation, mixing, and construction processes [1], mainly including toxic substances such as CO, SO_X_, organic compounds, and aromatic substances. Asphalt fumes not only cause irreversible pollution to the atmosphere and environment but also pose serious health risks to construction workers and surrounding residents [2,3,4]. Therefore, reducing flue gas emissions during asphalt pavement construction and ensuring the high quality of the finished pavement are pressing engineering challenges in the asphalt industry.

Currently, the main approach to improving the performance and reducing the toxic fume emissions of asphalt pavement is through optimizing pavement structure and materials. The most commonly used technique is incorporating flame-retardant and smoke-suppressant materials to enhance the flame resistance of asphalt pavement [5,6,7,8,9]. Traditional smoke suppressants like polymers, flame retardants, and warm mix additives often suffer from poor smoke suppression or reduced pavement performance. In contrast, nanomaterial smoke suppressants, such as nano-clay, offer both effective smoke suppression and improved asphalt performance. The layered structure of OMMT can effectively block the evaporation of lightweight components in asphalt, reducing smoke emissions. OMMT can also form intercalated or exfoliated structures with asphalt, enhancing its overall performance and showing promising application and development potential. However, research on the smoke suppression performance and mechanisms of OMMT-modified asphalt is still in its early stages and lacks theoretical support, limiting the development of OMMT as a smoke suppressant. Additionally, studies indicate that single OMMT-modified asphalt still has performance deficiencies, greatly restricting its engineering application. Therefore, the development and use of composite-modified asphalt have emerged as a key research focus. This study is significant for improving asphalt pavement performance and longevity, as well as advancing environmentally friendly asphalt solutions.

In this study, OMMT (organophilic montmorillonite) was chosen to modify SBS (styrene–butadiene–styrene) block copolymer-modified asphalt through composite modification, to prepare a nanocomposite OMMT/SBS modified asphalt with excellent smoke suppression performance and comprehensive road performance. Macroscopic and microscopic methods were employed to systematically investigate the rheological properties and the aging resistance of the nanocomposite OMMT/SBS-modified asphalt. During the service life of a composite-modified asphalt pavement, its mechanical properties are highly sensitive to variations in temperature and load frequency, which can affect the pavement’s performance [10]. Under varying temperatures and load frequencies, there are significant differences in its viscoelasticity and rheological properties. Under low temperature and high-speed loading conditions, asphalt tends to behave as an elastic solid. However, under high temperatures and low-frequency long-term loading conditions, the viscous fluid nature of asphalt becomes more apparent, as seen in the rutting of asphalt pavements during hot summer weather [11]. Asphalt aging is the process in which the mechanical structure and chemical composition of asphalt change due to external environmental factors such as heat and oxygen during construction and prolonged use, leading to a deterioration in its performance [12,13].

This study investigates the dynamic flow properties of OMMT/SBS composite-modified asphalt under various temperatures and load frequencies. Rheological tests were conducted on four asphalt samples (4%S, 4%S+1%O, 4%S+3%O, and 4%S+5%O), to examine the high- and low-temperature rheological performance of the modified asphalt and to understand how different OMMT contents affect its rheological behavior. DSR and FTIR tests were performed on the four modified asphalt samples before and after aging to analyze the impact of short-term and long-term aging on their rheological properties and chemical composition. The aging resistance of the composite modified asphalt was evaluated using rheological performance indicators (the complex modulus aging index and the phase angle aging index) and chemical functional group indicators (the carbonyl index growth rate and the sulfoxide index growth rate). This study will provide valuable insights for pavement and environmental researchers on improving asphalt pavement performance and longevity, as well as on the development of eco-friendly asphalt pavements.

## 2. Materials and Experiments

### 2.1. Materials

#### 2.1.1. Base Asphalt

The detailed test results of basic physical property indicators of asphalt can be found in Table 1.

#### 2.1.2. SBS

The main technical indicators of SBS are shown in Table 2.

#### 2.1.3. OMMT

The main technical indicators of OMMT are shown in Table 3.

**Figure 1:**
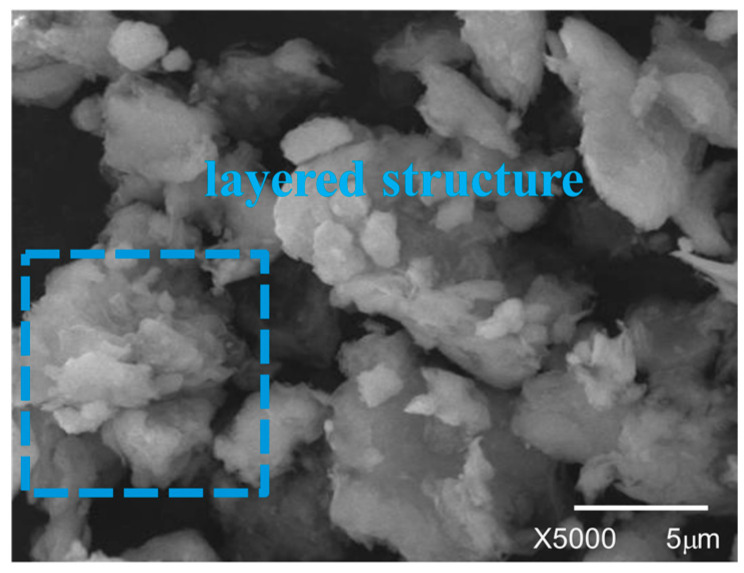
The SEM picture of OMMT.

Scanning electron microscopy (SEM, Hitachi S-3400N, Hitachi, Limited, Tokyo, Japan) was used to test and characterize the microscopic surface structure of the nano-clay. The results are shown in Figure 1. It can be observed that OMMT exhibits a layered structure. Many scholars’ studies have indicated that the lamellar structure of OMMT can be uniformly inserted into the asphalt system, forming a stable intercalated or exfoliated structure [14].

### 2.2. Preparation of OMMT/SBS Modified Asphalt

SBS-modified asphalt with a 4% SBS content was prepared and then modified with different amounts of OMMT (0%, 1%, 3%, and 5%). This process produced four distinct OMMT/SBS composite-modified asphalt samples, with the specific formulations outlined in Table 4.

**Figure 2:**
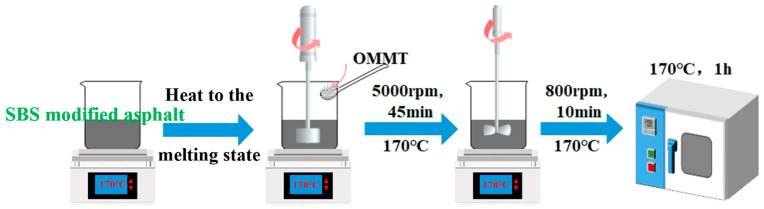
Preparation process of nano-clay/SBS modified asphalt.

Asphalt was sheared using a high-speed shear emulsifier, and SBS-modified asphalt was prepared by the melt blending method. The preparation process is illustrated in Figure 2, with the detailed operational steps as follows.

(1) Place the appropriate amount of grade 70 base asphalt into an oven, set the temperature to 140 °C, and heat until the asphalt is melted;

(2) Place the container with the asphalt into an electric heating jacket to ensure the asphalt can undergo high-speed shearing at a constant temperature. Set the temperature of the electric heating jacket to 170 °C. When the thermometer inserted into the asphalt reaches 170 °C, add 4% by mass of the SBS modifier. Shear at 2000 rpm for 20 min to allow it to fully dissolve in the asphalt;

(3) Adjust the shearing rate to 5000 rpm, add sulfur powder stabilizer, and shear at a high speed for 90 min;

(4) Stir with a low-speed mixer for 10 min to remove air bubbles from the asphalt;

(5) Place the sheared SBS-modified asphalt into a 170 °C oven for 1 h for aging, then cool to room temperature for subsequent testing.

Using the melt intercalation method to prepare nano-OMMT/SBS composite-modified asphalt, the preparation process is shown in Figure 2, and the specific operational steps are as follows.

(1) Before shearing, heat the OMMT in a 105 °C oven for 2 h to completely evaporate moisture;

(2) Place the SBS-modified asphalt prepared indoors in a 170 °C oven and heat to a molten state;

(3) Add a certain mass fraction (0%, 1%, 3%, 5%) of OMMT into the SBS-modified asphalt and shear at 5000 rpm for 45 min;

(4) Stir with a low-speed mixer for 10 min to remove air bubbles from the asphalt;

(5) Place the sheared OMMT/SBS composite-modified asphalt into a 170 °C oven for 1 h for aging, then cool to room temperature for subsequent testing.

### 2.3. Testing Program

The rheological tests of 4%S, 4%S+1%O, 4%S+3%O, and 4%S+5%O asphalt samples were carried out by a dynamic shear rheometer (DSR) and a bending beam rheometer (BBR) to investigate the high- and low-temperature rheological properties of the modified asphalts and how different OMMT contents affect the rheological performance of the modified asphalts.

#### 2.3.1. Temperature Sweep Test

Temperature scanning tests were conducted using a DSR to investigate the rheological properties of 4% SBS-modified asphalt and three different concentrations of nano-OMMT/SBS composite-modified asphalts. The study focused on how the OMMT content affects the complex modulus G* of SBS-modified asphalt at various temperatures. The tests utilized parallel plates with a diameter of 25 mm and a gap of 1 mm. The frequency was set to 1.59 Hz (10 rad/s), with a temperature range of 46~88 °C and a temperature increment of 6 °C.

#### 2.3.2. Frequency Sweep Test

Frequency sweep tests were performed on the modified asphalt using a DSR to examine its high-temperature rheological properties under varying load frequencies. The frequency range for the tests was 0.01 to 10 Hz, conducted at a temperature of 64 °C, with a controlled strain of 12%.

#### 2.3.3. MSCR Test

The creep stress level of the MSCR test was 0.1 kPa and 3.2 kPa, and 10 cycles of cyclic test were carried out with 10 s as a period. The test temperature was 64 °C, the diameter of parallel plates was 25 mm, the spacing was 1 mm, and the load frequency was 1.59 Hz. Four mechanical properties can be obtained by an MSCR test; namely, J_nr_, R, R_diff,_ and J_nr-diff_. J_nr_ can characterize the unrecoverable deformation of modified asphalt under repeated stress load, and the greater J_nr_ is, the worse the resistance of asphalt to permanent deformation is. R can evaluate the elastic recovery ability of asphalt under stress load. The stress sensitivity coefficient R_diff_ and J_nr-diff_ can evaluate the stress sensitivity of asphalt. The higher the value, the higher the sensitivity of asphalt to stress, and the more easily its mechanical properties are affected by stress.

#### 2.3.4. BBR Test

The low-temperature rheological properties of SBS-modified asphalt and OMMT/SBS-modified asphalt were evaluated using a bending beam rheometer. The asphalt beam specimens (125 mm × 12.5 mm × 6.25 mm) were submerged in an anhydrous ethanol solution, and a constant load of 0.98 N was applied at the midpoint of the beam supported by two points. The tests were conducted at temperatures of −12 °C, −18 °C, and −24 °C.

#### 2.3.5. Experimental Method of Asphalt Aging

(1) Short-term aging test

A thin film oven test (TFOT) was used to simulate the short-term aging of asphalt. The asphalt sample was prepared for 50 g, the temperature of the thin film oven was set to 163 °C for 5 h, and the sample dish was taken out after the test.

(2) Long-term aging test

To simulate the long-term aging of asphalt, a pressure aging vessel (PAV) test was conducted. In this test, 50 g of asphalt samples, which had been aged using a rolling thin film oven test (RTFOT), were placed in the PAV. The aging conditions were set at 100 °C, with a pressure of 2.1 MPa ± 0.1 MPa, for a duration of 20 h. After the aging process, the samples were removed from the aging vessel for further analysis.

#### 2.3.6. Infrared Spectroscopy Test

The effects of aging on the functional groups of 4%S, 4%S+1%O, 4%S+3%O, and 4%S+5%O asphalt samples were investigated by infrared spectroscopy.

#### 2.3.7. Atomic Force Microscope Test

AFM tests were performed on 4%S+3%O and 4%S asphalt samples before and after aging to analyze the effect of aging on the micromorphology of OMMT/SBS composite-modified asphalt and the effect of OMMT on the micromorphology of SBS-modified asphalt.

(1) AFM sample preparation

**Figure 3:**
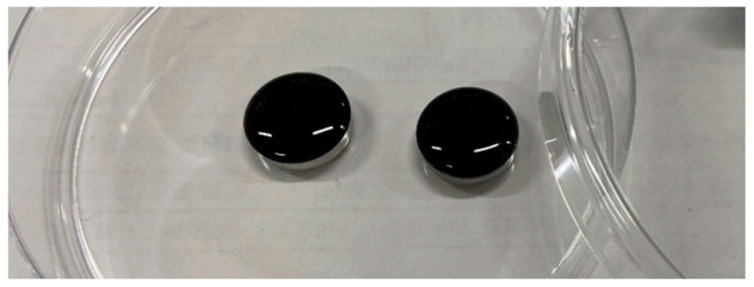
Testing sample of AFM.

The modified asphalt heated to a molten state was dropped into a small stainless steel container, as shown in Figure 3, and the sample was cooled at room temperature for AFM testing; during the whole process, attention needs to be paid to dust, and the sample surface should be ensured to be smooth and free of bubbles.

## 3. Results and Discussion

### 3.1. High-Temperature Performance

#### 3.1.1. Analysis of Temperature Scan Test Results

**Figure 4:**
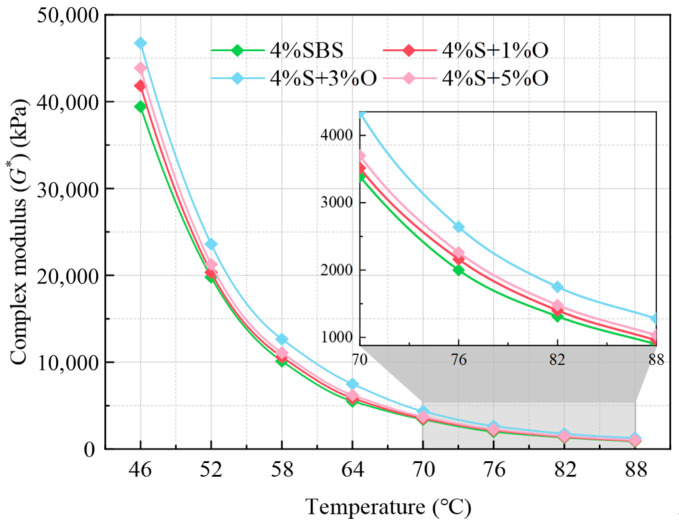
Complex modulus of four groups of modified asphalt.

The rheological property of OMMT/SBS-modified asphalt was evaluated through the temperature scanning test. The complex modulus G* test results are shown in Figure 4. G* can represent the shear deformation resistance of asphalt. The larger the G* of asphalt, the stronger the shear deformation resistance. It can be seen from the figure that with the increase in temperature, the characteristics of the asphalt viscous fluid become obvious, the G* of the four groups of asphalt gradually decreases, and the high-temperature rheological property decreases accordingly. This is determined by the different viscoelastic properties of asphalt under different temperature conditions, which also verifies that asphalt is temperature-dependent and very sensitive to temperature changes. The complex modulus of SBS-modified asphalt increased in the range of 46~88 °C after the addition of different amounts of OMMT modifier, that is, compared with OMMT/SBS-modified asphalt, SBS-modified asphalt was more prone to deformation under the same stress level. The results show that OMMT can improve the high-temperature shear deformation resistance of SBS-modified asphalt to a certain extent. This is due to the intercalation nanocomposite structure formed inside OMMT/SBS composite modified asphalt, which can enhance the stability of the three-dimensional structure of asphalt and prevent the dislocation of asphalt molecular chains under external forces, thus improving the modulus and strength of asphalt. The order of complex modulus of the four groups of modified asphalt is 4%S+3%O > 4%S+5%O > 4%S+1%O > 4%S+1%O > 4%S. Among them, the complex modulus of 4%S+3%O is significantly higher than that of the other three groups of asphalt, and there is little difference between the complex modulus of 4%S+1%O and 4%S+5%O. The increase in the complex modulus of 4%S+3%O is the largest, which increased by 18.5~42.3%. The increases of 4%S+1%O and 4%S+5%O were only 6.1–6.6% and 11.3–15.1%, respectively, which indicates that 3%OMMT has the most obvious effect on the high-temperature deformation resistance of SBS-modified asphalt.

**Figure 5:**
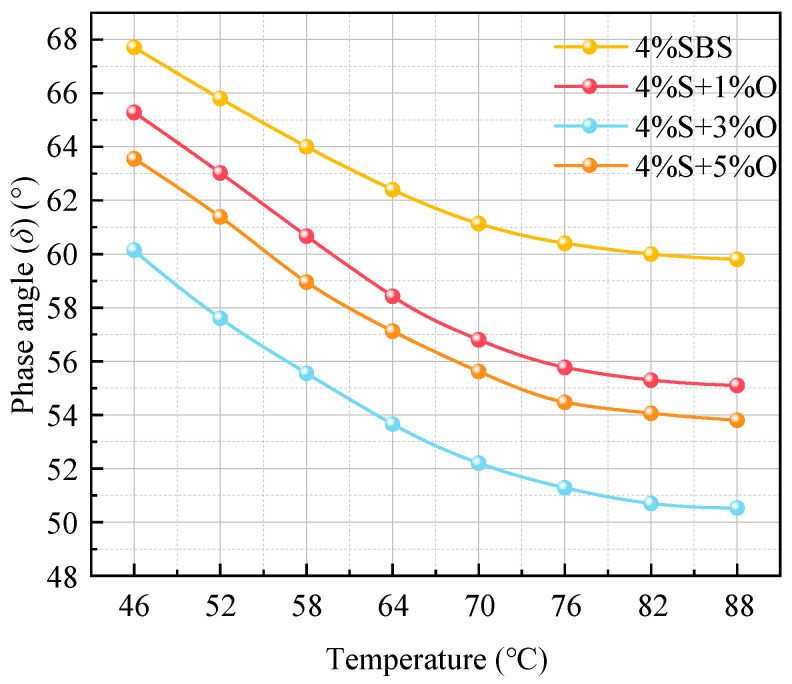
Phase angle of four groups of modified asphalt.

The angle *δ* test results of the four groups of bitumen at different temperatures are shown in Figure 5. The phase angle can evaluate the ratio of the viscous and elastic components of the bitumen, and measure the recoverable and unrecoverable deformation of the bitumen [15]. The smaller the phase angle, the more elastic components of the bitumen, and the more resistant to permanent deformation. Different from the general law that the phase angle increases with the increase in temperature, the phase angle of the four groups of bitumen in the figure all decreases with the increase in temperature. This may be due to the cross-linking between SBS and asphalt components, forming a spatial network cross-linking structure, and the enhanced intermolecular force of asphalt can maintain its viscous and elastic components to a certain extent [16]. In addition, nano-OMMT can improve the deformation resistance of SBS-modified asphalt, and the OMMT/SBS composite modification changes the viscoelastic properties of modified asphalt, resulting in a downward trend in the increase in the phase angle. In addition, with the addition of the OMMT modifier, the phase angle of SBS-modified asphalt decreases and the flow deformation resistance increases. When the OMMT content is 3%, the phase angle decreases most obviously. When the OMMT content is 1% and 5%, the phase angle of the two groups of modified bitumen is close.

**Figure 6:**
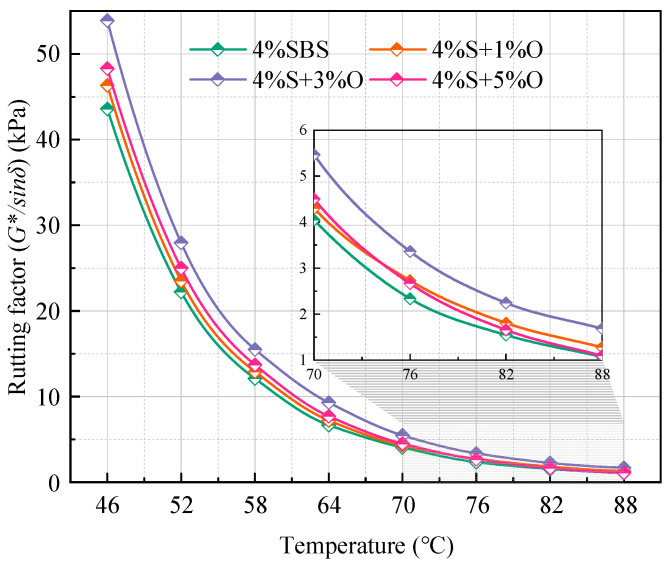
Rutting factor of four groups of modified asphalt.

Figure 6 shows the rutting factor test results of four groups of modified asphalt at 46~88 °C. As can be seen from the figure, after adding OMMT, the G*/sinδ of composite-modified asphalt is greater than that of 4%SBS-modified asphalt, and the G*/sinδ of 4%S at 46~88 °C is increased by different amounts of nano-OMMT, of which 3%OMMT has the most obvious effect on the G*/sinδ of 4%S. However, the enhancement effect of 1%OMMT and 5%OMMT is small, and the G*/sinδ of 4%S+5%O is lower than 4%S+1%O at 76~88 °C. The above results indicate that OMMT can enhance the rutting resistance of modified asphalt, that is, OMMT/SBS-modified asphalt is less prone to permanent deformation under the same load. This is due to the intercalation structure formed by the composite-modified asphalt, which enhances the intermolecular force of the asphalt so that the asphalt is not easily deformed under the action of external forces [17]. The high-temperature rutting resistance of 4%S+3%O is the best, which is the same as that of the complex modulus and phase angle. By observing the test results of the complex modulus, the phase angle, and the rutting factor, a common phenomenon can be found, that is, when the test temperature reaches 82 °C, the G*, *δ,* and G*/sinδ of the four groups of modified bitumen tend to be stable, indicating that the bitumen is close to becoming a viscous fluid at this temperature.

At the test frequency of 10 rad/s, asphalt can be divided into different performance grades (PGs) by using the critical temperature of G*/sinδ ≥ 1 kPa of unaged asphalt [18]. It can be seen in Figure 6 that the G*/sinδ of the four groups of bitumen at 88 °C is all greater than 1, indicating good high-temperature performance. To more accurately obtain the critical temperature corresponding to G*/sinδ ≥ 1 kPa, the rut factor log temperature is fitted by linear regression, and the linear regression equation is shown in Equation (1).

(1) $$\mathrm{lg}(G^{*}/sin\delta )=aT+b$$

where G*/sinδ is the rut factor, kPa; *a* is the slope (constant); *T* is the test temperature, °C; and *b* is the intercept (constant).

**Figure 7:**
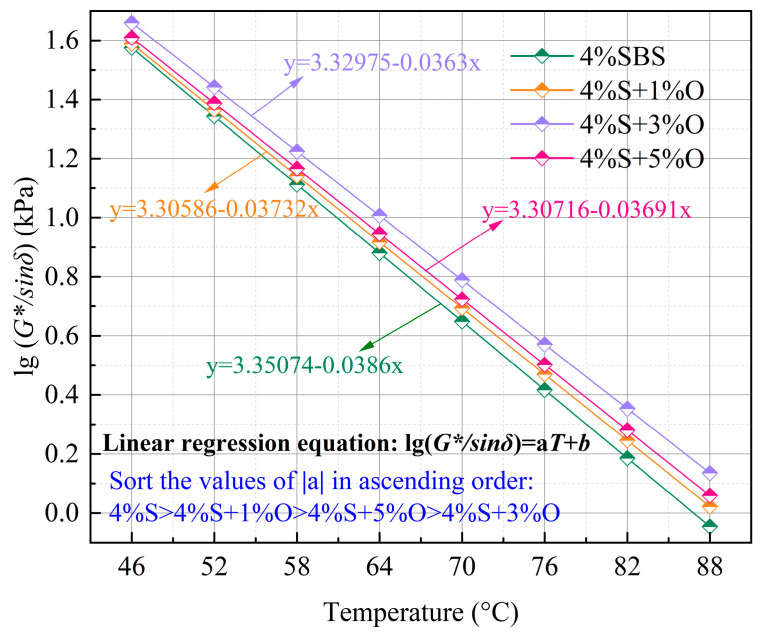
Rutting factor fitting.

The log temperature fitting results of the rutting factor are shown in Table 5 and Figure 7. The correlation coefficients R^2^ of the fitting of the four groups of original asphalt are all above 0.99, indicating a high degree of fitting. The linear regression equation can more accurately describe the change of the rutting factor with temperature. |a| is the absolute value of the slope of the fitting equation, which can be used to characterize the temperature-sensing performance of the asphalt material. The larger |a| is, the higher the temperature sensitivity of the asphalt material is, the more sensitive it is to temperature change, and the worse the high-temperature stability of the asphalt is. On the contrary, the smaller |a| is, the less sensitive the asphalt material is to temperature changes, and the better the high-temperature stability. According to Table 5, among the four types of modified asphalt, the 4%S modified asphalt has the largest |a| value, It can be seen from Table 5 that |a| is the largest for SBS-modified asphalt, indicating if its high-temperature stability is worse, the addition of OMMT can improve its temperature-sensing performance and enhance its high-temperature stability. An |a| of 4%S+3%O is the smallest, indicating that this asphalt has the strongest stability under high-temperature conditions. It can also be found from Table 5 that the critical temperature of the four groups of asphalt is contrary to |a|. The critical temperature of SBS-modified asphalt is the lowest, and that of 4%S+3%O is the highest. Generally speaking, the higher the critical temperature, the stronger the high-temperature rutting resistance of asphalt materials. Compared with SBS-modified asphalt, OMMT/SBS-modified asphalt has better high-temperature rutting resistance. By comparing the fitting equations of the four groups of modified bitumen in Figure 7, it can be seen that the G*/sinδ of 4%S+3%O is the highest at different temperatures, which indicates that 4%S+3%O has the best high-temperature rutting resistance.

#### 3.1.2. Analysis of Frequency Sweep Test Results

The high-temperature rheological properties of OMMT/SBS-modified asphalt under different load frequencies were studied by a frequency sweep test.

(1) Time-temperature equivalence principle

Asphalt is a typical viscoelastic material, and its mechanical behavior is strongly dependent on both time and temperature. Asphalt materials can exhibit the same mechanical behavior under the action of an equivalent temperature and time, which is called the time–temperature equivalence principle [19]. Asphalt can show the same mechanical behavior under a high temperature, high-speed load and a low temperature, long time load. Based on the time–temperature equivalence principle, the mechanical properties of asphalt in a wider temperature range and frequency range can be accurately predicted and described [20,21,22]. The calculation formula of the asphalt time–temperature equivalence principle is shown in Equations (2) and (3).

(2) $$\left|G^{*}\left(T,\omega \right)\right|=\left|G^{*}\left(T_{0},\omega_{r}\right)\right|$$

(3) $$\omega_{r}=\omega \cdot a(T)$$

where *T* is the test temperature (°C); *ω* is the test angular frequency (rad/s); |G*(*T,ω*)| is the G* (Pa) at test temperature and angular frequency; *T_0_* is the reference temperature (°C); *ω_r_* is the Reduced angular frequency (rad/s); |G*(*T_0_,ω_r_*)| is the G* (Pa) measured at the reference temperature and reduced angular frequency; and *a(T)* is the shift factor.

Based on the time–temperature equivalence principle, a specific reference temperature is selected, and then the complex modulus curve or phase angle curve at other temperatures is translated to the curve at the reference temperature, thus forming a fitted complex modulus main curve or phase angle main curve [23]. During the formation of the main curve, the distance from the curve at other temperatures to the curve at the reference temperature is called the shift factor *a(T)*, which can be calculated according to the WLF equation, and its formula is shown in Equation (4).

(4) $$\mathrm{lg}a\left(T\right)=\frac{-C_{1}\left(T-T_{0}\right)}{C_{2}+\left(T-T_{0}\right)}$$

where *C*_1_ and *C*_2_ are material parameters, reflecting the temperature sensitivity of asphalt materials, and can refer to the experience value.

(2) Principal curve analysis

**Figure 8:**
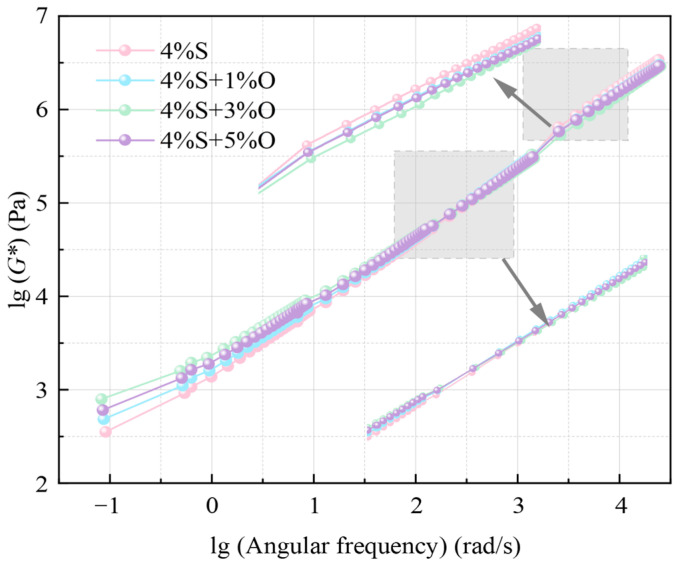
Main curve of complex modulus.

In this study, 60 °C was selected as the reference temperature, and the complex modulus main curves of four groups of modified asphalt were obtained by fitting according to the time–temperature equivalent principle, as shown in Figure 8 (The gray areas are magnified sections of the figure where the lines are close together). It can be seen from the figure that the main curves of the complex modulus of the four groups of bitumen are similar in shape, close to linear, and the changing trend is consistent. The complex modulus increases with the increase in angular frequency, that is, in the low-temperature and high-frequency region, the modified bitumen has stronger shear deformation resistance. This is in line with the viscoelastic characteristics of asphalt. Under the action of a low temperature and a high-speed load, the elastic characteristics of asphalt are more obvious, and it is not easy to produce shear deformation. The coincidence degree of the main curve is higher in the high-frequency region, but the difference is obvious in the low-frequency region. In the high-temperature and low-frequency region, the order of the complex modulus of the four groups of modified asphalt is as follows: 4%S+3%O > 4%S+5%O > 4%S+1%O > 4%S, which indicates that OMMT can improve the high-temperature shear deformation resistance of SBS-modified asphalt, and the deformation resistance of 4%S+3%O at high temperature is the strongest, which is the same as the complex modulus test results of temperature scanning. With the increase in the loading frequency, the main curves of the complex modulus of the four groups of modified asphalt gradually approach and overlaps occur in the frequency range of 10^2^~10^3^. In the low temperature and high-frequency region, the complex modulus of OMMT/SBS-modified asphalt is lower than that of SBS-modified asphalt, which indicates that when the temperature decreases to a certain extent, the asphalt tends to behave like an elastic solid, and the addition of OMMT modifier actually reduces the low-temperature deformation resistance of SBS-modified asphalt.

**Figure 9:**
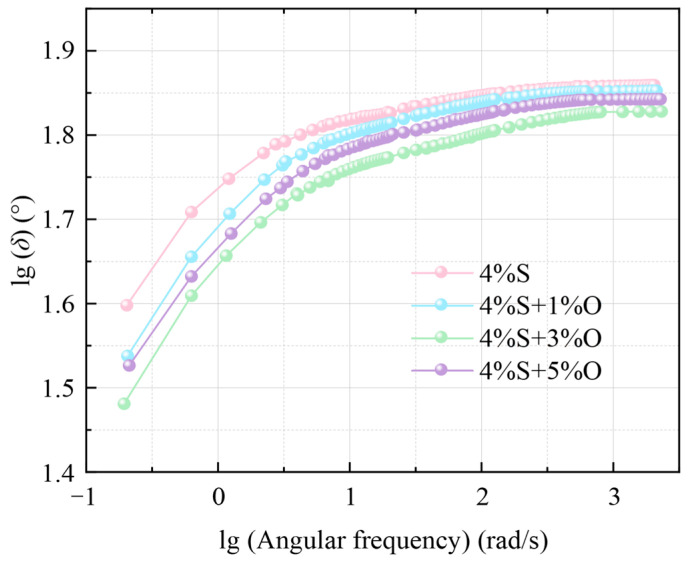
Main curve of phase angle.

The main curve of the phase angle of the four groups of modified bitumen is similar in shape and has the same change trend, as shown in Figure 9. As the frequency increases, the phase angle of modified bitumen also gradually increases; at this time, modified bitumen presents more viscous characteristics and a less elastic composition. At the same frequency, the order of phase angle of the four groups of modified asphalt is as follows: 4%S > 4%S+1%O > 4%S+5%O > 4%S+3%O, indicating that OMMT/SBS composite-modified asphalt is less prone to deformation than 4%S under the same load frequency, and that OMMT/SBS-modified asphalt has the strongest deformation resistance when the OMMT content is 3%. In the high-frequency region above 10^3^ rad/s, the asphalt tends to be an elastic solid material, and the phase angle of the four groups of modified asphalt tends to be stable.

#### 3.1.3. Analysis of MSCR Test Results

**Figure 10:**
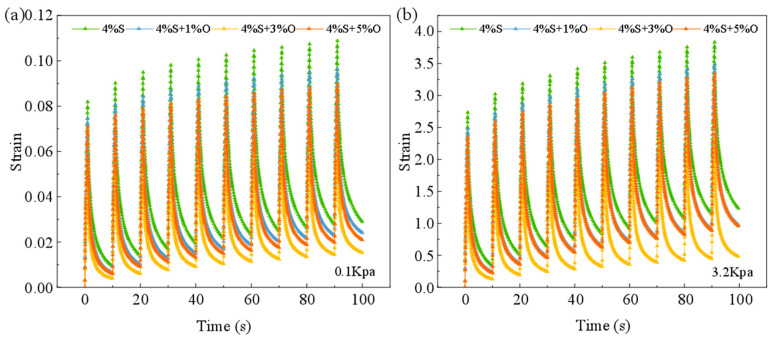
Strain–time diagram: (**a**) 0.1 kPa; (**b**) 3.2 kPa.

The MSCR test results for four types of asphalt under stress levels of 0.1 kPa and 3.2 kPa yield strain–time curves, as shown in Figure 10a,b. From these figures, it can be observed that, under different stress levels, the cumulative non-recoverable strain for all asphalts exhibits a similar trend, with increasing strain over time. Additionally, the higher the applied shear stress, the greater the cumulative strain in the modified asphalt. For the same type of asphalt, the cumulative non-recoverable strain under 3.2 kPa of stress is significantly higher than that under 0.1 kPa of stress, indicating that higher shear stress leads to larger and less recoverable strains. Under both 0.1 kPa and 3.2 kPa loads, SBS-modified asphalt shows the highest non-recoverable residual strain, indicating that the nano-OMMT modifier can improve the high-temperature creep performance of the modified asphalt and enhance its resistance to flow deformation at high temperatures. This improvement is due to the insertion structure formed by OMMT in the asphalt, which effectively hinders the movement of asphalt molecular chains, thereby enhancing the high-temperature performance of the modified asphalt. At both stress levels, the strain for 4%S+3%O is less than that for 4%S+1%O and 4%S+5%O, suggesting that an optimal amount of OMMT has the most significant effect on improving the flow deformation resistance of the modified asphalt.

**Figure 11:**
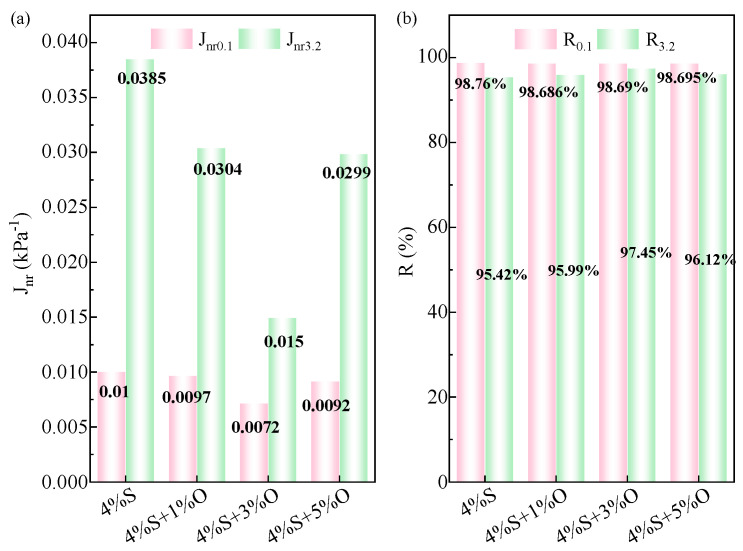
J_nr_ and R of modified asphalt at 0.1 kPa and 3.2 kPa: (**a**) J_nr0.1_ and J_nr3.2_; (**b**) R_0.1_ and R_3.2_.

The MSCR test results for modified asphalt at 64 °C, showing J_nr_ and R, are depicted in Figure 11. Figure 11a presents the non-recoverable shear modulus J_nr_ for four types of modified asphalt. It can be seen from the figure that as the stress level increases, the J_nr_ of the asphalt material becomes larger, indicating a poorer high-temperature rutting resistance and a higher tendency for permanent deformation. The test results show that the J_nr_ of SBS-modified asphalt is the largest at the same stress level, which reflects that the energy loss of SBS-modified asphalt in the creep process is large, and it is easy to produce large cumulative deformation and forms permanent ruts under a high temperature and load. It also shows that the high-temperature rutting resistance of SBS-modified asphalt is significantly improved after OMMT modification. At the stress levels of 0.1 kPa and 3.2 kPa, J_nr_ of OMMT/SBS-modified asphalt changes in the same way, that is, with the increase in OMMT modifier content, J_nr_ first decreases and then increases, among which J_nr_ of 4%S+3%O is the smallest. Compared with SBS-modified asphalt, the J_nr_ of OMMT/SBS-modified asphalt is the smallest. The J_nr0.1_ and J_nr3.2_ values decreased by 28.6% and 61.1%, respectively, which indicates that the high temperature rutting resistance of 4%S+3%O under load is the most prominent, and the permanent deformation is also the smallest. At a higher load stress level (3.2 kPa), the change amplitude of J_nr_ is also larger and more obvious, which indicates that the OMMT modifier has a more significant effect on the high-temperature resistance to permanent deformation of SBS-modified asphalt under heavy traffic. Figure 11b shows the calculation results of four groups of modified asphalt R. As can be seen from the figure, the higher the stress level, the smaller the R, the worse the elastic recovery ability, and the worse the high-temperature performance, which is exactly the opposite trend of J_nr_. The R_0.1_ and R_3.2_ of the four groups of modified bitumen are high, indicating that the modified bitumen prepared in this study has a strong elastic recovery ability. In addition, when the stress level is high, the R of the four modified bitumen groups is small, and the elastic recovery ability is poor. Under the creep stress levels of 0.1 kPa and 3.2 kPa, the R of SBS-modified asphalt is the smallest among the four groups, indicating that the OMMT modifier can improve the elastic recovery ability of SBS-modified asphalt. When the load stress is 0.1 kPa, the R of the four groups of modified bitumen is very high and the difference is very small, which is close to 99%, indicating that the four groups of modified bitumen have excellent creep properties at high temperatures under low loads. When the load stress is 3.2 kPa, the change of R of the four groups of modified asphalt is more obvious. With the increase in OMMT content from 1% to 5%, R increases slightly at first and then decreases slightly, in which the R of 4%S+3%O is the largest, indicating that its elastic recovery ability is strong. The variation law of modified asphalt R under a 3.2 kPa load stress level is different from that under 0.1 kPa of stress, which indicates that the OMMT modifier has a more obvious effect on the high-temperature deformation resistance of asphalt under heavy traffic, which is consistent with the law shown by J_nr_.

**Figure 12:**
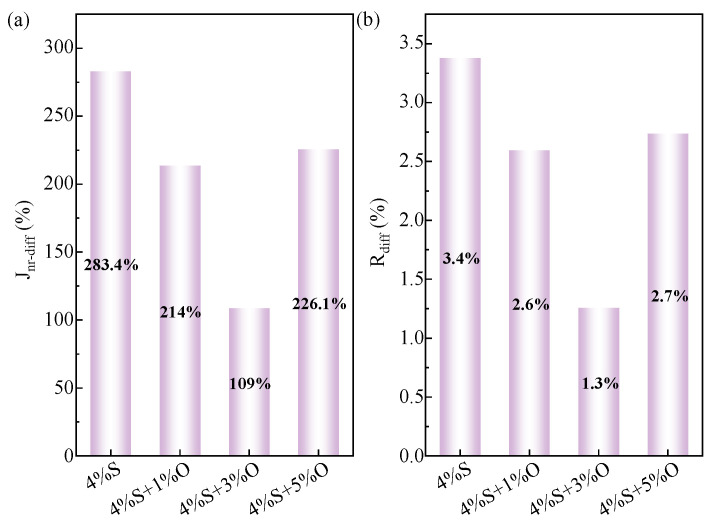
J_nr-diff_ and R_diff_ of four groups of modified asphalt: (**a**) J_nr-diff_; (**b**) R_diff_.

Stress sensitivity is an index reflecting the degree of influence of stress on asphalt rheological properties, which will affect the service quality and stability of asphalt pavement [24]. The more sensitive the modified asphalt is to load stress, the easier it is to deform. The J_nr-diff_ and R_diff_ of SBS-modified asphalt and OMMT/SBS-modified asphalt at 64 °C are shown in Figure 12. As can be seen from the figure, J_nr-diff_ and R_diff_ change in the same law, SBS-modified asphalt showed the highest sensitivity to stress, 4%S+1%O, 4%S+3%O, and 4%S+5%O J_nr-diff_ and R_diff_ were lower, and the incorporation of OMMT improved the stress sensitivity of SBS-modified asphalt. This is because the asphalt will expand when heated, the internal void will increase, the intermolecular force will weaken, and the intermolecular arrangement will not be tight enough, resulting in deformation under the action of stress. After OMMT modification, the compatibility between SBS and asphalt is enhanced, and a good spatial network crosslinked structure is formed inside the modified asphalt, and OMMT and SBS modified asphalt form an intercalated nanocomposite structure. The synergistic effect of these two structures has a good effect on the thermal movement of the asphalt molecular chain, so that the molecular arrangement is closer, and the relative displacement between the asphalt molecules does not easily occur, which effectively improves the thermal stability of the asphalt so that the high-temperature performance of the modified asphalt is improved.

### 3.2. Low-Temperature Rheological Properties

**Figure 13:**
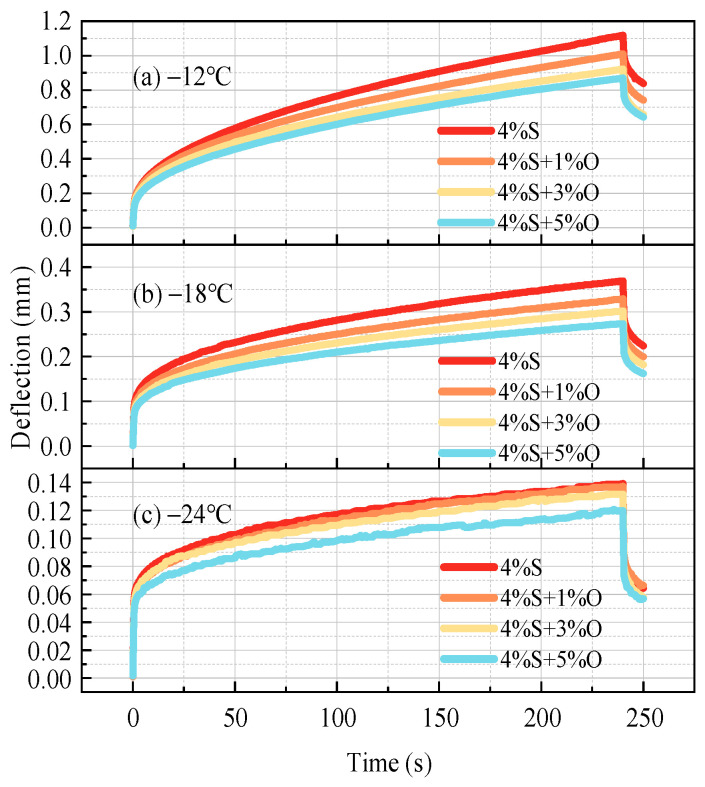
Time–deflection curves at −12 °C, −18 °C, and −24 °C.

The low-temperature rheological properties of modified asphalt were analyzed by a BBR test. Figure 13 shows the time–deflection variation curves of four groups of modified asphalt trabeculars at −12 °C, −18 °C and −24 °C during the BBR test. The smaller the deflection value, the smaller the degree of deformation that the modified asphalt can withstand during the loading process, the worse its flexibility, and the more prone it is to brittle fracture. The larger the deflection value, the greater the degree of deformation that the modified asphalt can withstand during the loading process, the less prone to brittle fracture, and the better the low-temperature cracking resistance of the modified asphalt is. As can be seen from Figure 13, the time–deflection curves of SBS-modified asphalt and OMMT/SBS-modified asphalt show the same trend of change, and the asphalt deformation gradually increases with the increase in load time. Under the same temperature and load time, the deflection values of the four groups of modified asphalt are as follows: 4%SBS > 4%SBS+1%OMMT > 4%SBS+3%OMMT > 4%SBS+5%OMMT, that is, the low-temperature crack resistance of the four groups of modified asphalt deteriorates in turn. The deflection value of 4%SBS modified asphalt is the largest and its low-temperature cracking resistance is the best. However, after the addition of the nano-OMMT modifier, the deflection value of modified asphalt becomes smaller and the low-temperature cracking resistance becomes worse, and the deflection value of modified asphalt becomes smaller and smaller with the increase in OMMT content. The results show that OMMT will lead to low-temperature performance attenuation of modified asphalt, and the higher the OMMT content, the more obvious the low-temperature performance attenuation.

**Figure 14:**
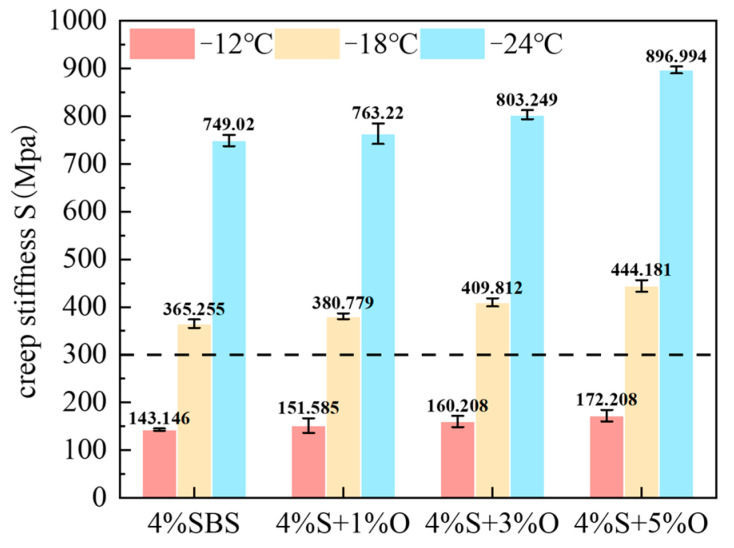
Creep stiffness of modified asphalts.

**Figure 15:**
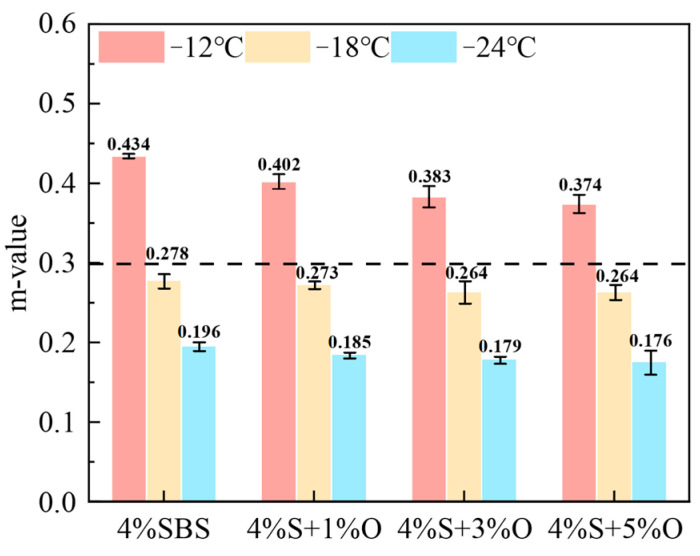
m of modified asphalts.

The creep stiffness S reflects the ability of asphalt material to resist load deformation at low temperatures, and the creep stiffness m reflects the time sensitivity and stress relaxation characteristics of the creep stiffness. The larger the S of asphalt material, the smaller the m, which means that the flexibility of asphalt material is worse, the time sensitivity of asphalt is also greater, and the stress relaxation property is worse. The smaller S is, the larger m is, which indicates that the low-temperature cracking resistance and stress relaxation performance of asphalt materials are better. To ensure a good low-temperature performance of asphalt pavement, AASHTO test regulations stipulate that the S of asphalt material should be lower than 300 MPa and the m should be greater than 0.3 [25]. The test results of the creep stiffness S and the creep rate m of SBS-modified asphalt and OMMT/SBS-modified asphalt at −12 °C, −18 °C, and −24 °C and during 60 s under load are shown in Figure 14 and Figure 15. As can be seen from the figures, if S of 4%S is the smallest and m is the largest, its low-temperature cracking resistance is the best; however, after the addition of a nano-modifier, the S of 4%S increases and m decreases, which indicates that OMMT will cause the low-temperature performance of SBS-modified asphalt to deteriorate, and the higher the OMMT content, the more serious the low-temperature performance attenuation of composite modified asphalt will be. The S and m of the four groups of modified asphalt all meet the specification requirements of S < 300 MPa and m > 0.3 at −12 °C, that is, the low-temperature grade of the four groups of modified asphalt is −12 °C, which indicates that the addition of an OMMT modifier will not affect the low-temperature PG grade of the asphalt material, but will affect the flexibility of the asphalt material. The lower the temperature, the greater the degree of influence.

### 3.3. Aging Resistance

#### 3.3.1. Analysis of Test Results

**Figure 16:**
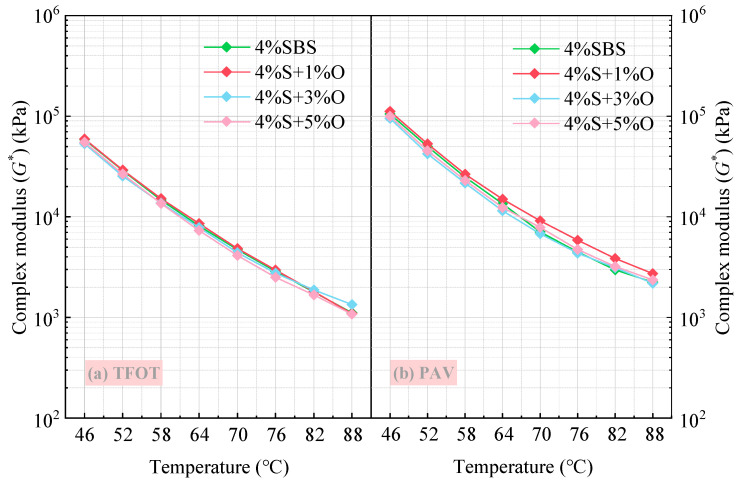
Complex modulus of modified asphalt after (**a**) TFOT and (**b**) PAV.

Temperature scanning tests were conducted on 4%S, 4%S+1%O, 4%S+3%O, and 4%S+5%O after aging. The influence of the degree of non-assimilation on the complex modulus of the modified asphalt is shown in Figure 16. It can be seen that the complex modulus of the four groups of modified asphalt increases to different degrees after short-term aging and long-term aging. The light components or oxidized into heavy components cause the asphalt to harden. Moreover, the increase in complex modulus becomes obvious with the deepening of the aging degree of modified asphalt. After short-term aging, the complex moduli of 4%S, 4%S+1%O, 4%S+3%O, and 4%S+5%O are increased by about 24~49%, 15~42%, 5~16%, and 8~25%, respectively, at different temperatures. Among them, the complex modulus of 4%SBS-modified asphalt has the largest change amplitude before and after short-term aging, and that of 4%S+3%O has the smallest change amplitude. After long-term aging under different temperatures, the complex moduli of 4%S, 4%S+1%O, 4%S+3%O, and 4%S+5%O are increased by about 150–166%, 168–185%, 71–104%, and 99–128%, respectively, in which the complex moduli of 4%S+1%O changes the most. The change of 4%S+3%O is still the smallest.

**Figure 17:**
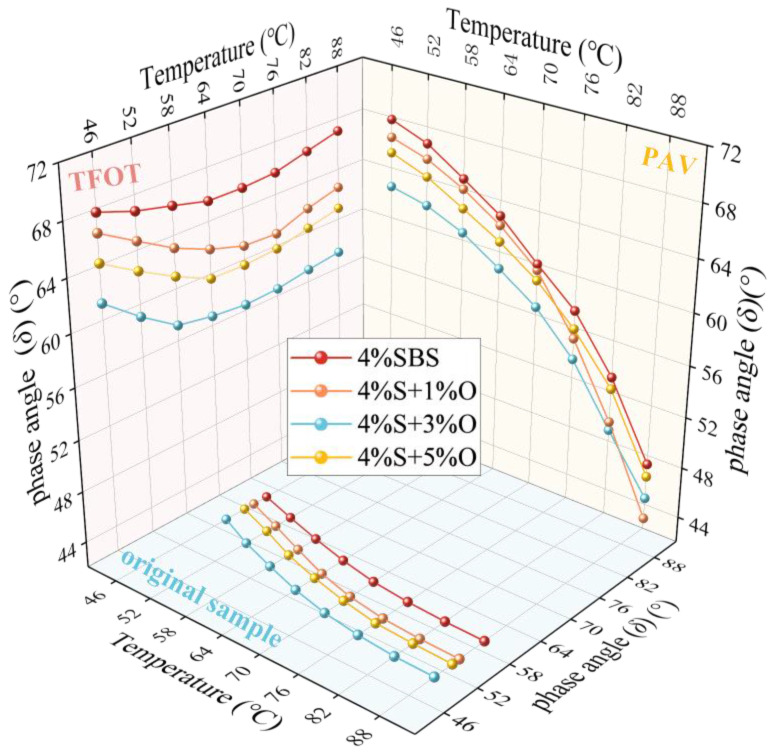
Phase angle of modified asphalt before and after aging.

**Figure 18:**
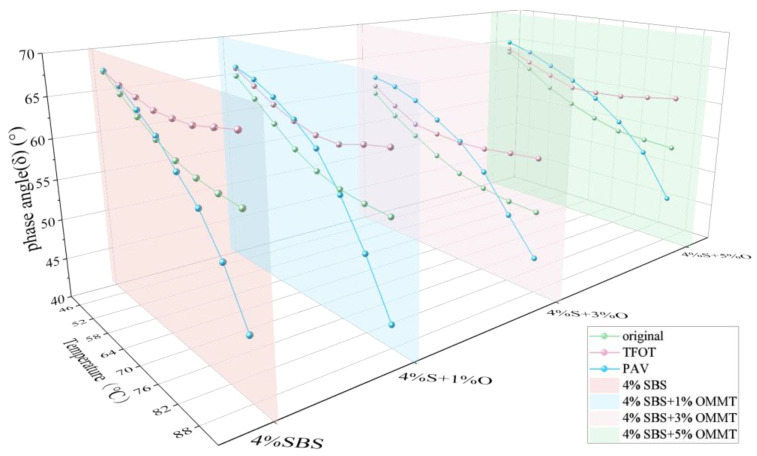
Effect of aging on the phase angle of modified asphalt.

The phase angle test results of the temperature scan test before and after aging are shown in Figure 17 and Figure 18. It can be seen from Figure 17 that the trend of the phase angle curve after aging is slightly different from that of the original modified asphalt. The trend of the phase angle curve of the original modified asphalt is that the phase angle decreases with the increase in temperature. After short-term aging, the phase angle decreases first and then increases with the increase in temperature. After long-term aging, the phase angle decreases significantly with the increase in temperature, and the decline rate is greater than that of the original asphalt, which indicates that the temperature sensitivity of SBS-modified asphalt and OMMT/SBS-modified asphalt is enhanced after long-term aging. In addition, there is a special phenomenon worth discussing. As shown in Figure 18, the four groups of modified asphalt do not decrease but increase after short-term aging, which is different from the influence trend of aging on the asphalt phase angle commonly found in academic papers. Generally speaking, asphalt will harden after aging, and the elastic components in asphalt will increase, resulting in a decrease in the asphalt phase angle. In this study, however, the opposite is true. After the long-term aging of modified asphalt, the phase angle of the original modified asphalt first increases and then decreases.

**Figure 19:**
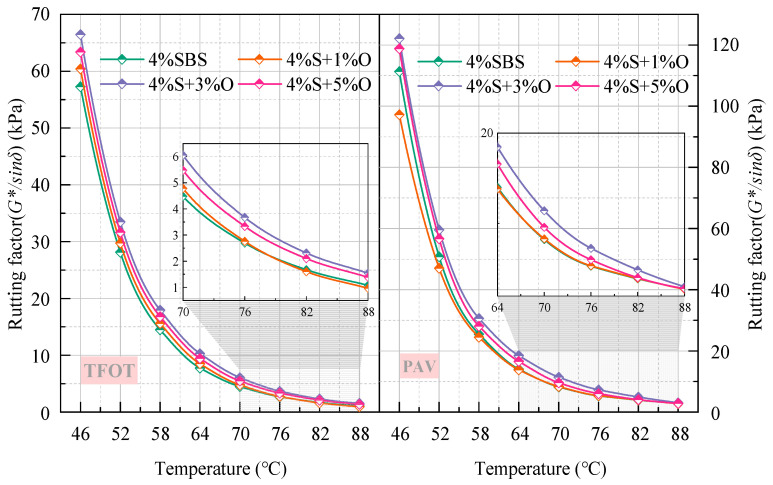
Rutting factor of modified asphalt after TFOT and PAV.

The effects of short-term and long-term aging on SBS-modified asphalt and the G*/sinδ of OMMT/SBS-modified asphalt are shown in Figure 19 (The gray areas are magnified sections of the figure where the lines are close together). It can be seen from the figure that after aging, the G*/sinδ of the four groups of modified asphalt is increased to different degrees, and the higher the aging degree, the greater the increase of G*/sinδ. The order of G*/sinδ size of the four groups of modified bitumen is 4%S+3%O > 4%S+5%O > 4%S+1%O > 4%S. The experimental results show that the G*/sinδ of SBS-modified asphalt increases after short-term aging with OMMT, and OMMT can improve the high temperature rutting resistance of asphalt after short-term aging, in which the G*/sinδ of 4%S+3%O is the largest and the most resistant to permanent deformation. In other words, when the OMMT content is 3%, it has the most significant improvement effect on the high-temperature rutting resistance of asphalt after short-term aging. The American SHRP specification stipulates that the G*/sinδ of asphalt after short-term aging is not less than 2.2 kPa.

The G*/sinδ of the four groups of modified asphalt is greater than 2.2 kPa at 76 °C, while the G*/sinδ of the four groups of modified asphalt is less than 2.2 kPa at 82 °C. After long-term aging, the G*/sinδ of the four groups of modified bitumen is further increased, and the order is 4%S+3%O > 4%S+5%O > 4%S > 4%S+1%O. The test results show that the G*/sinδ of OMMT/SBS-modified asphalt is still higher than that of SBS-modified asphalt when the dosage is 3% and 5%. OMMT can improve the permanent deformation resistance of SBS-modified asphalt after long-term aging and improve the high-temperature performance of SBS-modified asphalt. When the OMMT content is 1%, the G*/sinδ of 4%S+1%O is smaller than that of SBS-modified asphalt, but OMMT weakens the high-temperature permanent deformation resistance of SBS-modified asphalt. These results indicate that an appropriate OMMT level can effectively improve the high-temperature rutting resistance of SBS-modified asphalt after aging.

#### 3.3.2. Aging Index of Complex Modulus

The calculation formula of *CAI* of the complex modulus aging index is shown in Equation (5). The larger *CAI* is, the more serious asphalt aging is, and asphalt has improved aging resistance.

(5) $$CAI=\frac{G^{*}}{G_{0}^{*}}$$

where *CAI* is the aging index of complex modulus; G^*^ is the complex modulus of asphalt after aging; and $G_{0}^{*}$ is the complex modulus of the original asphalt.

**Figure 20:**
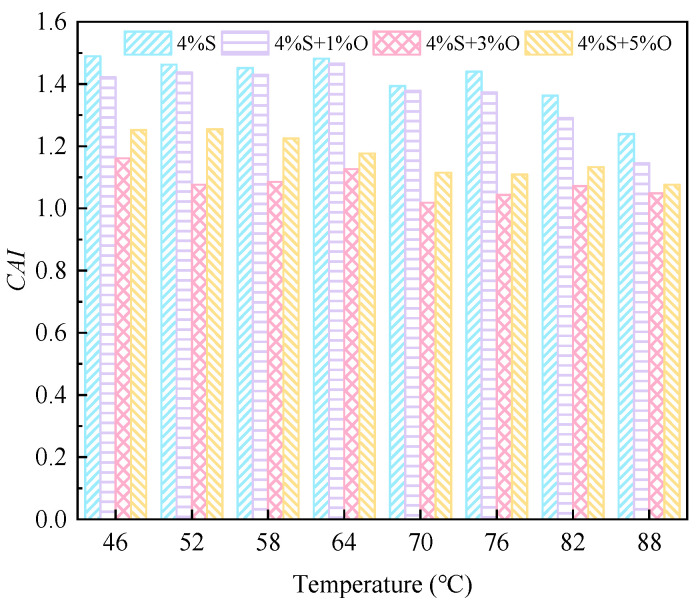
*CAI* after short-term aging.

**Figure 21:**
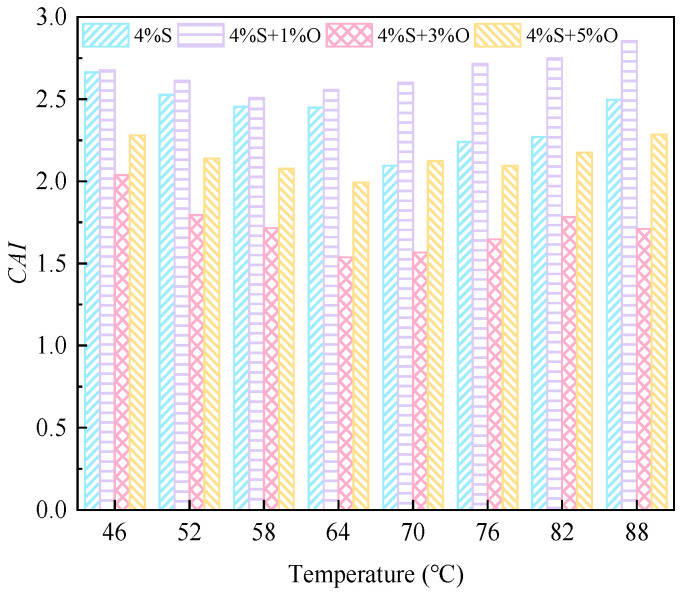
*CAI* after long-term aging.

The aging index of the complex modulus after short-term aging and long-term aging is shown in Figure 20 and Figure 21. As can be seen from Figure 20, the aging index of the complex modulus of the four groups of modified asphalt after short-term aging is ranked as 4%S > 4%S+1%O > 4%S+5%O > 4%S+3%O in the full range of temperature range, which is consistent with the changing trend of the increased amplitude of the complex modulus of the four groups of asphalt. This ranking indicates that OMMT can reduce the short-term aging degree of SBS-modified asphalt under the same environmental conditions and improve the aging resistance of SBS-modified asphalt. The aging resistance of OMMT/SBS-modified asphalt does not increase with the increase in OMMT content, among which the aging index of 4%S+3%O is the smallest. The aging resistance is also the best, indicating that 3% of OMMT has the most significant effect on the aging resistance of SBS-modified asphalt. It also shows that an appropriate amount of OMMT can improve the aging resistance of SBS-modified asphalt to the greatest extent. The order of the complex modulus aging index of the four groups of modified asphalt after long-term aging is different from that of short-term aging in the whole temperature range, and the order is 4%S+1%O > 4%S > 4%S+5%O > 4%S+3%O. After long-term aging, 1% of OMMT could not improve the aging resistance of SBS-modified asphalt, but the aging resistance of 4%S+3%O and 4%S+5%O was still better than that of SBS-modified asphalt, and 4%S+3%O had the smallest complex modulus aging index and the best long-term aging resistance. These results indicate that an appropriate OMMT level can improve the long-term aging resistance of SBS-modified asphalt.

#### 3.3.3. Phase Angle Aging Index

The calculation formula of the phase angle aging index *PAI* is shown in Equation (6). The smaller the *PAI* is, the more serious asphalt aging is, and the asphalt has poor aging resistance.

(6) $$PAI=\frac{\delta }{\delta_{0}}$$

where *PAI* is the phase angle aging index; *δ* is the aged asphalt phase angle; and *δ*_o_ is the phase angle of the original asphalt.

**Figure 22:**
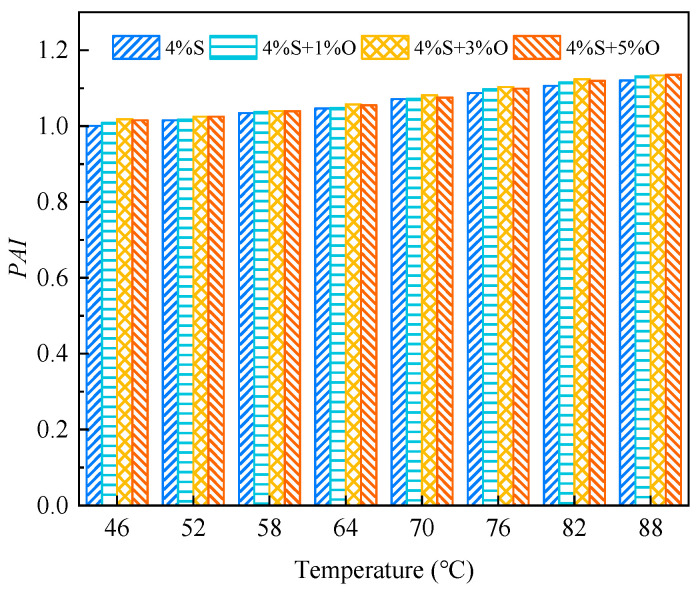
*PAI* after short-term aging.

**Figure 23:**
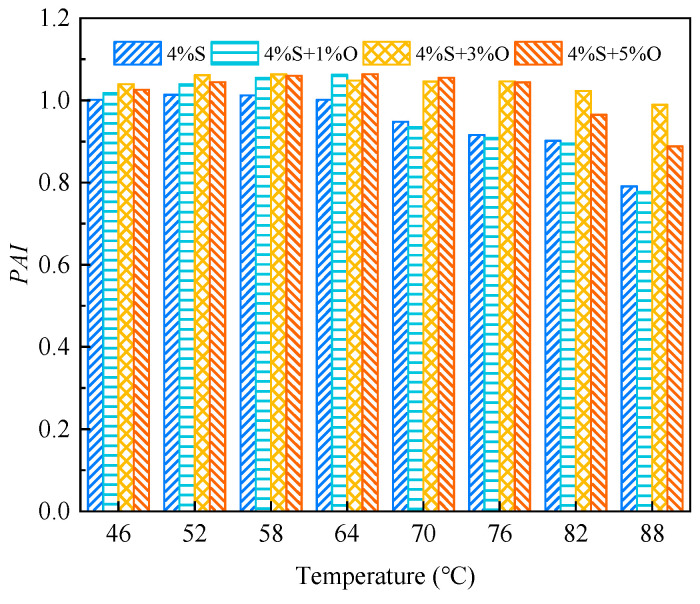
*PAI* after long-term aging.

The phase angle aging index of modified asphalt after aging is shown in Figure 22 and Figure 23. It can be seen from the figures that the change in the phase angle aging index of the four groups of modified asphalt is not very obvious; especially, the phase angle aging index after short-term aging has little difference. After short-term aging and long-term aging, the phase angle aging index of 4%+3%O is the largest, and it can be seen that 4%S+3%O has the best aging resistance.

#### 3.3.4. Effect of Aging on Functional Groups of Modified Asphalt

**Figure 24:**
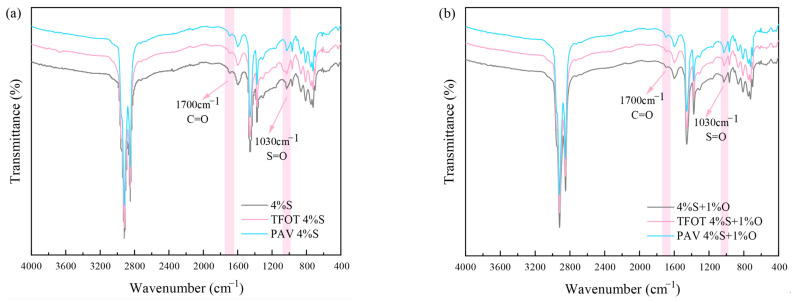

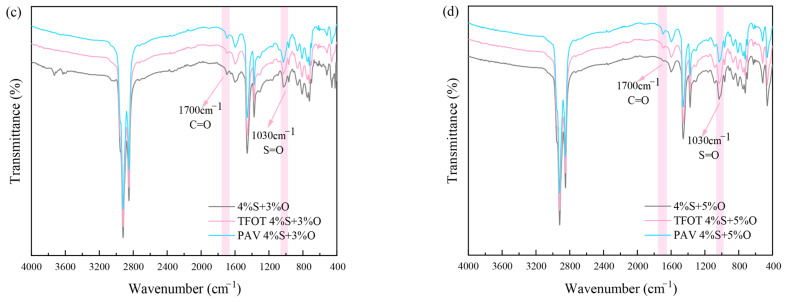
FTIR spectrum of aged and unaged modified asphalt: (**a**) 4%S; (**b**) 4%S+1%O; (**c**) 4%S+3%O; and (**d**) 4%S+5%O.

Some relevant studies have shown that the aging resistance of asphalt can be more comprehensively evaluated according to the rheological properties of asphalt before and after aging [26]. The aging resistance of modified asphalt was only compared from the perspective of macroscopic rheological properties. However, the aging of asphalt not only leads to the decay of macroscopic mechanical properties of asphalt but also changes the chemical composition of asphalt. Therefore, it is not only necessary to study the aging resistance of modified asphalt according to the changes in physical rheological properties before and after aging but also to conduct a more in-depth analysis from the microchemical level. The infrared spectrum test results of aging and non-aging 4%S, 4%S+1%O, 4%S+3%O, and 4%S+5%O are shown in Figure 24.

The aging of asphalt is the process of the oxidation of asphalt. Carbonyl C=O and Sulfoxyl S=O are both oxygen-containing polar functional groups closely related to the aging behavior of asphalt. C=O is formed by the oxidation of hydrocarbons such as unsaturated carbon chains, and S=O is formed by the oxidation of sulfide [27,28]. Therefore, the degree of aging and the aging resistance of asphalt are usually characterized according to the changes of C=O and S=O before and after aging. The infrared spectra of the four groups of aged and non-aged modified asphalt are shown in Figure 24. It can be observed that the C=O and S=O absorption peaks of 4%S, 4%S+1%O, 4%S+3%O, and 4%S+5%O all have certain changes after aging. In this study, the C=O index (*CI*) and S=O index (*SI*) were used to quantify and characterize the content of C=O and S=O in asphalt. The larger the *CI* and *SI*, the higher the content of C=O and S=O. The *CI* growth rate (*I_C=O_*) and the *SI* growth rate (*I_S=O_*) were used to quantify the aging degree and aging resistance of modified asphalt. OMNIC software (OMNIC9.2, Thermo Nicolet, Waltham, MA, USA) was used to calculate the absorption peak area. The calculation formulas of *CI* and *SI* are shown in Equations (7) and (8), and the calculation formulas of *I_C=O_* and *I_S=O_* are shown in Equations (9) and (10).

(7) $$CI=\frac{A_{c=o}}{\sum A_{2000{\mathrm{cm}}^{-1}\sim 600{\mathrm{cm}}^{-1}}}$$

(8) $$SI=\frac{A_{s=o}}{\sum A_{2000{\mathrm{cm}}^{-1}\sim 600{\mathrm{cm}}^{-1}}}$$

(9) $$I_{c=o}=\frac{CI_{aged}-CI_{unaged}}{CI_{unaged}}\times 100\%$$

(10) $$I_{s=o}=\frac{SI_{aged}-SI_{unaged}}{SI_{unaged}}\times 100\%$$

where *A_C=O_* is the C=O absorption peak area; *A_S=O_* is the S=O absorption peak area; $\sum A_{2000{\mathrm{cm}}^{-1}\sim 600{\mathrm{cm}}^{-1}}$ is the sum of the absorption peak areas in the range of 12,000 cm^−1^~600 cm^−1^ wave number; *CI_aged_*, *CI_unaged_* is the C=O index after aging and the C=O index without aging; and *SI_aged_*, *SI_unaged_* is the S=O index after aging and the S=O index without aging.

**Figure 25:**
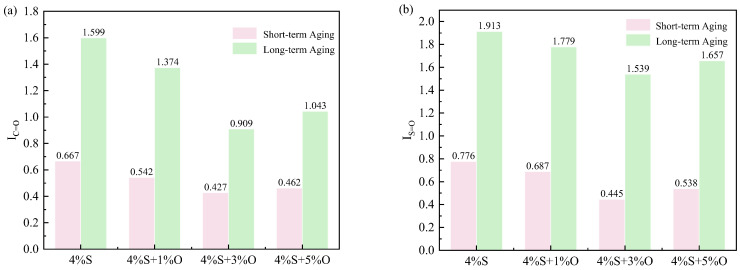
(**a**) *I_C=O_* of modified asphalt; (**b**) *I_S=O_* of modified asphalt.

The *CI* and *SI* calculation results of the four modified bitumen groups of 4%S, 4%S+1%O, 4%S+3%O, and 4%S+5%O are shown in Table 6 and Table 7. It can be seen from the table that the *CI* and *SI* of the four groups of modified asphalt have the same change law, that is, the *CI* and *SI* of the modified asphalt increase with the deepening of aging degree. Moreover, after the PAV test, the *CI* and *SI* values of the modified asphalt are significantly higher than those of the modified asphalt after the TFOT test, which is because the asphalt is subjected to the high temperature and high pressure of the pressure aging vessel for a long time, and the asphalt matrix is heated and expanded. The intermolecular arrangement is not tight enough, and it is easier for oxygen to penetrate the asphalt matrix, resulting in the oxidation of unsaturated carbon chains to produce C=O, and the oxidation of sulfide to produce S=O, so the *CI* and *SI* after long-term aging are greatly increased. After adding OMMT, the *CI* and *SI* of 4%S decreased, indicating that OMMT could inhibit the oxidation reaction in the asphalt matrix and reduce the formation of C=O and S=O. Based on the *CI* and *SI*, *I_C=O_* and *I_S=O_* are calculated, and the results are shown in Figure 25. As can be seen from the figure, *I_C=O_* and *I_S=O_* of 4%S decrease after the addition of OMMT, indicating that OMMT slows down the aging degree of modified asphalt, and the aging resistance of composite modified asphalt is better than 4%S. When the content of OMMT IS 3%, the *I_C=O_* and *I_S=O_* of OMMT/SBS composite-modified asphalt are the lowest, indicating that 4%S+3%O has the best aging resistance. The same impact trend is also obtained by analyzing the aging resistance of modified asphalt according to the change in rheological properties. Considering the economic benefit and the performance of the modified asphalt, the optimal mixing amount of nano-OMMT/SBS composite-modified asphalt can be determined as 4%SBS+3%OMMT. 

#### 3.3.5. Microscopic Morphology Analysis of Modified Asphalt during the Aging Process

**Figure 26:**
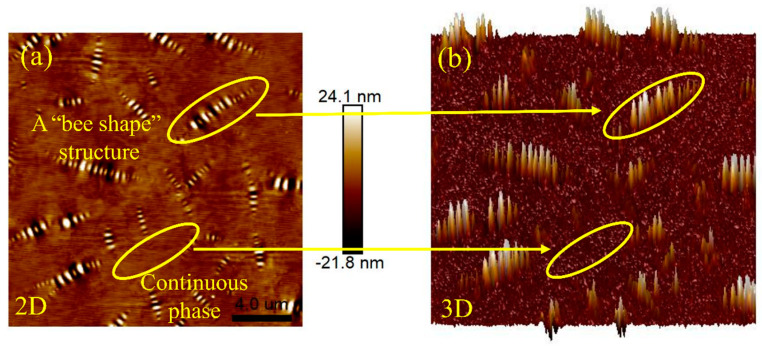
Micro morphology of 4%S (**a**) 2D; (**b**) 3D.

**Figure 27:**
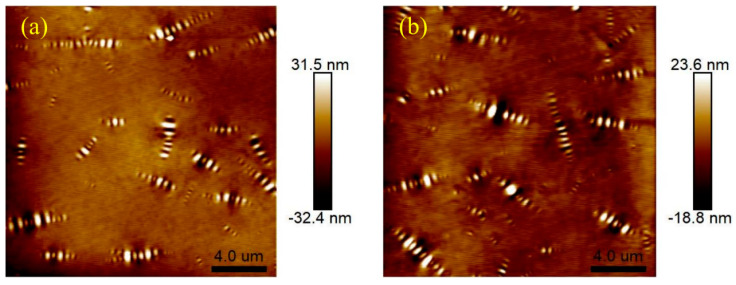
Micro morphology of 4%S: (**a**) after TFOT; (**b**) PAV.

Atomic force microscopy (AFM) tests were conducted on 4%S+3%O and 4%S before and after aging. Figure 26 shows the two-dimensional and three-dimensional microscopic morphology of 4%S. The striped structure with alternating light, dark, yellow, and white in the figure is called the “bee shape” because it resembles a bee. Asphalt has an obvious two-phase structure, namely continuous phase and dispersed phase. The color of the continuous phase is darker and its height is lower, while the color of the dispersed phase is brighter and its height is higher than the continuous phase, and the “bee shape” structure is the dispersed phase. In addition, a large number of studies have shown that the formation of a “bee shape” structure is mainly due to the aggregation and precipitation of microcrystalline wax and asphaltene in asphalt components, and the molecular properties of asphaltene and the surrounding asphalt are quite different. Microcrystalline wax is wrapped in asphaltene to form crystals and precipitates outward, forming a “bee shape” structure dispersed in the continuous phase of the asphalt [29,30]. Figure 27 shows the microscopic morphology of 4%S after TFOT and PAV testing. By comparing the morphology of 4%S without aging with that of 4%S after aging, it can be found that after the aging of 4%S, the contrast of the dispersed phase and continuous phase decreases, and the boundary definition of the “bee shape” structure decreases and becomes blurred. This shows that after aging, the asphalt molecules around the dispersed phase of the “bee” structure are associated and gradually develop into a single-phase structure.

**Figure 28:**
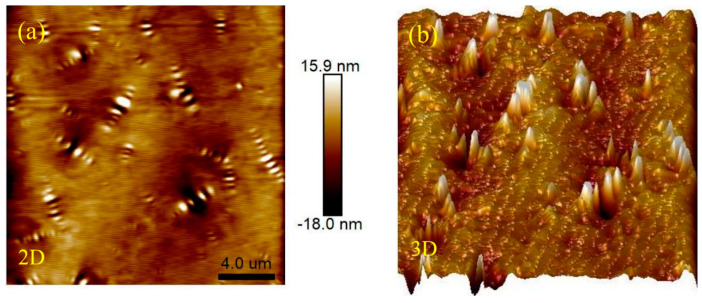
Micro morphology of 4%S+3%O: (**a**) 2D; (**b**) 3D.

**Figure 29:**
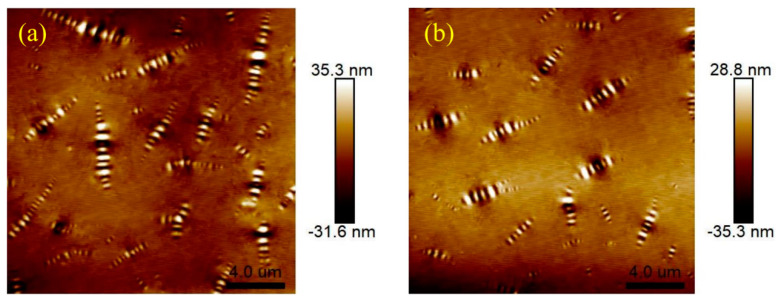
Micro morphology of 4%S+3%O: (**a**) after TFOT; (**b**) after PAV.

AFM scanning was performed on the unaged 4%S+3%O, and the microtopography obtained is shown in Figure 28. Compared with the non-aging 4%S, the addition of OMMT reduces the contrast between the two phases, and the boundary between the “bee shape” structure and the continuous phase becomes blurred, which is not as clear as before OMMT modification, and in the three-dimensional topography of 4%S and 4%S+3%O, it can be found that the asphalt surface becomes uneven. The reason may be that OMMT mainly reacts with the continuous phase in the modified asphalt, increasing the stiffness of the continuous phase, making the stiffness of the continuous phase close to that of the dispersed phase, and the morphology of the modified asphalt develops into a single-phase system [31]. In addition, compared with 4%S, the number of “bee” structures in 4%S+3%O decreases, and the length and height of the bee structure decreases, but its width increases. Figure 29 shows the microscopic morphology of 4%S+3%O after TFOT and PAV testing. Comparing the morphology of 4%S+3%O without aging with that of 4%S+3%O after aging, it can be found that after aging, the contrast between the dispersed phase and continuous phase is improved, and the boundary between the “bee shape” structure and continuous phase becomes clear. The results show that OMMT can inhibit the dispersive phase association and single-phase tendency of modified asphalt in the aging process and improve the aging resistance of modified asphalt. This may be due to the good intercalation structure of OMMT/SBS modified asphalt, showing a good “oxygen inhibition” ability, which helps to inhibit the dispersed phase association and single-phase trend of modified asphalt in the aging process. Therefore, the aging resistance of modified asphalt is improved after OMMT modification.

## 4. Conclusions

This study conducted DSR tests and BBR tests on nano-OMMT/SBS composite-modified asphalt to evaluate its rheological properties. It explored the effects of aging on the rheological performance and chemical functional groups of the modified asphalt. The aging resistance of the composite-modified asphalt was assessed based on rheological parameters and chemical functional group indicators. The main conclusions are as follows. 

(1) OMMT can increase the complex modulus, rutting factor, and creep recovery rate of SBS-modified asphalt while reducing the phase angle, non-recoverable creep compliance, and stress sensitivity coefficient. This indicates that OMMT can decrease the sensitivity of modified asphalt to load stress and improve its high-temperature rheological performance. The optimal improvement effect is observed when the OMMT content is 3%.

(2) OMMT can significantly enhance the high-temperature rheological properties of SBS-modified asphalt. However, BBR test results show that after OMMT modification, the creep stiffness of SBS-modified asphalt increases, the creep rate decreases, the flexibility of asphalt material decreases, and the low-temperature cracking resistance decreases.

(3) After both short-term and long-term aging, the aging index of the complex modulus for OMMT/SBS composite-modified asphalt is lower than that of SBS-modified asphalt, while the phase angle aging index is higher. This indicates that OMMT can enhance the aging resistance of SBS-modified asphalt. Additionally, the effect of OMMT content on the aging resistance and high-temperature rheological properties of SBS-modified asphalt shows a common trend, and the performance improvement is most significant when the OMMT content is 3%.

(4) After adding OMMT, CI, SI, IC=O, and IS=O of 4%S decreased, indicating that OMMT could inhibit the oxidation reaction in the asphalt matrix and reduce the formation of C=O and S=O. OMMT slowed down the aging degree of modified asphalt and improved the aging resistance of modified asphalt.

In this study, numerous microscopic experiments were conducted, but the analyses of these microscopic experiments were independent and not interconnected. However, there may be correlations among these microscopic phenomena. Therefore, in future research, it would be beneficial to construct a microscopic evaluation system to analyze the correlations among various microscopic phenomena and indicators, exploring the reliability of their mutual representation and conducting a multidimensional analysis of microscopic mechanisms.

## Figures and Tables

**Table 1:** Basic technical indicators of 70# base asphalt.

Test Item	Result	Test Method
Penetration (25 °C, 100 g, 5 s), 0.1 mm	69	T0604
Ductility (10 °C, 5 cm/min), cm	21	T0605
Ductility (15 °C, 5 cm/min), cm	108	T0605
Softening point, °C	49	T0606
Rotary viscometers (135 °C), mPa·s	420	T0625
Solubility (trichloroethylene), %	99.7	T0607
Flash point, °C	278	T0611
Density (15 °C), g/cm^3^	1.036	T0603
Change of residue quality after TFOT, %	−0.15	T0609
Residual penetration ratio after TFOT, %	73	T0604
Residual ductility after TFOT (10 °C), cm	6.7	T0605
Residual ductility after TFOT (15 °C), cm	61	T0605

**Table 2:** Main technical indicators of SBS.

Test Item	Unit	Result
Block ratio	-	30/70
Volatile component	%	0.7
Ash	%	0.2
300% constant elongation stress	MPa	2.2
Tensile strength	MPa	16
Break stretch	%	750
Shore-hardness	-	71

**Table 3:** Main technical indicators of OMMT.

Test Item	Unit	Result
Appearance	-	White flowable powder
Volatile component	%	≤3.5
Granularity	%	≥98
Proportion	-	1.6
Bulk density	(kg/m^3^)	470
Loss on ignition	%	≤40

**Table 4:** Compound scheme of OMMT/SBS composite modified asphalt.

OMMT (%)	Designation (4% SBS)
0%OMMT	4%S
1%OMMT	4%S+1%O
3%OMMT	4%S+3%O
5%OMMT	4%S+5%O

**Table 5:** Fitting parameters.

Asphalt Type	|*a*|	Fitting Equation	R^2^	Critical Temperature (°C)
4%S	0.0386	y = 3.35074 − 0.0386x	0.996	86.81
4%S+1%O	0.03732	y = 3.30586 − 0.03732x	0.997	88.58
4%S+3%O	0.0363	y = 3.32975 − 0.0363x	0.997	91.73
4%S+5%O	0.03691	y = 3.30716 − 0.03691x	0.999	89.60

**Table 6:** Calculation result of *CI*.

Modified Asphalt Samples	Non-Aged	Short-Term Aging	Long-Term Aging
4%S	0.0162	0.027	0.0421
4%S+1%O	0.0131	0.0202	0.0311
4%S+3%O	0.011	0.0157	0.021
4%S+5%O	0.0093	0.0136	0.019

**Table 7:** Calculation result of *SI*.

Modified Asphalt Samples	Non-Aged	Short-Term Aging	Long-Term Aging
4%S	0.1421	0.2524	0.414
4%S+1%O	0.131	0.221	0.3641
4%S+3%O	0.115	0.1662	0.292
4%S+5%O	0.0891	0.137	0.2367

## Data Availability

The original contributions presented in the study are included in the article, further inquiries can be directed to the corresponding author.
